# Supplementary material for: Content-rich biological network constructed by mining PubMed abstracts
Source: BMC Bioinformatics. 2004 Oct 8;5:147. doi: 10.1186/1471-2105-5-147 (PMC528731; doi:10.1186/1471-2105-5-147)
Supplement: Additional File 2 — The original results of the above study (non-essential files are deleted to keep the file size under the limit set by BMC bioinformatics). [file 1471-2105-5-147-S2.bz2 › chilibotAdditionalFile2/dip10/581ID8624811E10949/html/B2M_HLA.html]

 


 **B2M** and **HLA** 
  
Found 815 abstracts in PubMed, retrieved 10.  
 

 What does Google say? 
 PDF only 
| .edu only 

---

**Interactive relationship** (e.g. stimulation, inhibition, etc)

**Neutral relationship**- Class I  **HLA**  oligomerization at the surface of B cells is controlled by exogenous beta 2 microglobulin  [ **B2M** ]  implications in activation of cytotoxic T lymphocytes.  Ref: 12618477 Int Immunol, 2003

**Non-interactive relationship** (e.g. studied together, co-existance, homology, etc.)

- Here, the sensitivity of this homotypic association to exogenous beta 2 microglobulin  [ **B2M** ]  beta 2 m and the role of free heavy chains FHC in class I  **HLA**  oligomerization were investigated on a B lymphoblastoid cell line, JY.  Ref: 12618477 Int Immunol, 2003
- The regions analyzed consisted of the  **HLA**  class I heavy chain genes located within the MHC genomic region on chromosome arm 6p, the  **HLA**  class I light chain beta 2 microglobulin  [ **B2M** ] ,  **B2M**  gene on chromosome arm 15q, and the putative  **HLA**  modifier of methylation gene MEMO1 located on chromosome arm 1q.  Ref: 12759931 Genes Chromosomes Cancer, 2003
- These cells were systematically characterized by Northern and or Western blotting e.g., mini transcriptome mini proteome analysis for the expression of  **HLA**  A, B, C, beta 2 microglobulin  [ **B2M** ] , and the members of the antigen processing machinery of class I molecules LMP2, LMP7, TAP1, TAP2, tapasin, calreticulin, calnexin, and ERp57.  Ref: 12874016 Cancer Res, 2003
- The expression patterns of delta, tapasin,  **HLA**  Class I antigen, beta 2 microglobulin  [ **B2M** ] , and Survivin did not demonstrate any significant association with the clinical course of disease.  Ref: 12712472 Cancer, 2003
- In contrast to the high frequency of APM component down regulation,  **HLA**  class I heavy chain and beta 2 microglobulin  [ **B2M** ]  defects were detected in only 12 and 10% of the lesions, respectively.  Ref: 12738726 Clin Cancer Res, 2003
